# Comparison of the prognostic value of platelet-based prognostic models in patients with malignant hepatic tumors after TACE therapy^1^

**DOI:** 10.1590/s0102-865020190070000010

**Published:** 2019-09-16

**Authors:** Qian Cheng Du, Chen Liang Hu, Yan Yan Wang, Ying Zhou

**Affiliations:** I Master, Department of General Surgery, Shanghai Fourth People's Hospital Affiliated to Tongji University School of Medicine, Shanghai, China. Intellectual, scientific, conception and design of the study; acquisition, analysis and interpretation of data; technical procedures; statistical analysis; manuscript preparation, final approval; II Master, Department of Hepatopancreatobiliary Surgery, the Affiliated Hospital of Qinghai University, Xining, China. Intellectual, scientific, conception and design of the study; acquisition, analysis and interpretation of data; technical procedures; statistical analysis; manuscript preparation, final approval; III Master, Department of Hematology, Affiliated Fuyang Hospital of Anhui Medical University, Fuyang, China. Intellectual, scientific, conception and design of the study; acquisition, analysis and interpretation of data; technical procedures; statistical analysis; manuscript preparation, final approval; IV Full Professor, Department of Hepatopancreatobiliary Surgery, the Affiliated Hospital of Qinghai University and Qinghai Province Key Laboratory of Hydatid Disease Research, Qinghai University, Xining, China. Acquisition, analypsis and interpretation of data; technical procedures; critical revision; manuscript preparation, final approval

**Keywords:** Transcatheter arterial chemoembolization, Blood Platelets, Liver Neoplasms, Prognosis

## Abstract

**Purpose:**

To investigate the prognostic value of 17 platelet-based prognostic scores in patients with malignant hepatic tumors after TACE therapy.

**Methods:**

In total, 92 patients were divided into death group and survival group according to long-term follow-up results. The AUC was calculated to determine the optimal cut-off values for predicting prognosis. To determine better prognostic models, platelet-based models were analyzed separately after being showed as binary according to cut-off values. Cumulative survival rates of malignant hepatic tumors were calculated using Kaplan-Meier curves and differences were analyzed by the log-rank test. Univariate and multivariate analyses were performed to identify platelet-based prognostic scores associated with overall survival.

**Results:**

Univariate analysis showed that APGA, APRI, FIB-4, FibroQ, GUCI, King's score, Lok index, PAPAS, cirrhosis, number of tumors, vascular cancer embolus, AFP, ALP and APTT were significantly related to prognosis. A multivariate analysis showed that the APGA, number of tumors, ALP and APTT were independently associated with overall survival.

**Conclusion:**

This study showed that the APGA, a platelet-based prognostic score, was an independent marker of prognosis in patients with malignant hepatic tumors after TACE and was superior to the other platelet-based prognostic scores in terms of prognostic ability.

## Introduction

Malignant hepatic tumors were the sixth most frequently diagnosed cancer worldwide, and the fourth leading cause of tumor-related deaths^1^. Patients with malignant hepatic tumors were treated by different methods based on TNM stage of tumor and hepatic function reserve before accepting treatment. Although the prognosis of malignant hepatic tumors had improved significantly, the prognosis was still unsatisfactory with a 5-year survival rate of about 5%-6%^2^. There were no obvious symptoms at the early stage, and most of the patients had entered the middle and late stage at the time of diagnosis. Patients with intermediate or advanced stage tumors were not suitable for radical treatment according to the Barcelona Clinic Liver Cancer staging system (BCLC)^3^. Transcatheter arterial chemoembolization (TACE) was the optimum palliative treatment for patients with unresectable malignant hepatic tumors. The median survival time of patients with malignant hepatic tumors after TACE was 12 months, which was significantly lower than that after radical liver resection^4^. Therefore, it is very important to evaluate the prognosis of patients performing TACE according to specific laboratory indexes before treatment.

The main functions of platelets (PLT) were hemostasis and thrombosis. Recently, many reports showed that platelets and platelet-activation molecules played an important part in tumor cell growth, metastasis, and angiogenesis^5^ ^–^ ^8^. Non-invasive predicting models based on laboratory tests had been widely used, and could be a feasible method for evaluating the extent of hepatic fibrosis, tumor recurrence and so on. The aspartate aminotransferase (AST)-to-platelet ratio index (APRI), the King's Score, the FibroQ and other models had been proposed to predict hepatic fibrosis, liver cirrhosis and liver dysfunction in patients with chronic hepatitis^9^ ^–^ ^11^. Hepatitis, cirrhosis and liver cancer were the three stages of disease development; however, it was not clear whether these models could predict the prognosis of malignant liver tumor. Maeda *et* al.^12^ described that Fibrosis-4 (FIB-4) index could evaluate the recurrence rate and 5-year recurrence rate after hepatectomy in patients with hepatocellular carcinoma. However, the non-invasive models based on tumor clinical characteristics and peritumoral fibrosis for predicting prognosis of patients accepting TACE were still rare, especially in overall survival.

We aimed to investigate the prognostic value of these platelet-based prognostic models in patients with malignant hepatic tumors who underwent TACE, and to select the most predictive model for guiding TACE preoperative evaluation. Thus, a retrospective analysis of 92 patients who had received TACE was performed.

## Methods

In total, 92 patients with intermediate or advanced diagnosed malignant hepatic tumors and performing TACE that had been treated at the Intervention Department, the Affiliated Hospital of Qinghai University, between November 2011 and October 2018 were enrolled in the study. Comprehensively baseline information, including clinical, laboratory, imageological and follow-up data was available for all patients. Patients with coexistent hematologic diseases, patients who had received blood transfusion within the previous 6 months, extrahepatic tumor, and incomplete data were excluded. Patients were followed after TACE treatment every 3-6 months until death or dropout. This study was approved by the Ethics Committee at the Qinghai University Affiliated Hospital and complies with the provisions of the Declaration of Helsinki.

The diagnosis of malignant hepatic tumors should be based on imageological techniques obtained by dynamic contrast-enhanced computed tomography (CT), dynamic contrast-enhanced magnetic resonance imaging (MRI), and the typical peripheral blood tumor markers of hepatic tumors^13^. Tumor-related parameters such as the maximal diameter of tumor, the number of tumors, vascular invasion, vascular cancer embolus, the diameter of spleen and extrahepatic metastases were evaluated by imageological techniques. All peripheral blood parameters were derived from blood draws taken within 7 days before the first TACE.

### Platelet-based prognostic scores and other variables

Electronic medical records were used to obtain relevant information, including age, sex, monocyte fraction, neutrophil fraction, levels of alanine aminotransferase (ALT), AST, total cholesterol (TCH), alkaline phosphatase (ALP), γ-glutamyl transpeptidase (GGT), PLT, prothrombin time (PT), activated partial thromboplastin time (APTT), international normalized ratio (INR), alpha-fetoprotein (AFP), etiology (HBV or HCV), ascites, cirrhosis status, number of tumors, diameter of maximal tumor, vascular invasion, vascular cancer embolus, diameter of spleen, extrahepatic metastases and concomitant disease. The primary outcome measure for the study was survival status (death or survival).

The Pohl score^14^, aspartate aminotransferase/alanine aminotransferase ratio-platelet count score (AARP)^15^, age/ platelet count index (API)^16^, cirrhosis discriminant score (CDS)^17^, APRI^9^, FIB-4^18^, FibroQ^11^, Fibrosis index based on the three factors (Lok index)^19^, Goteborg University cirrhosis index (GUCI)^20^, aspartateaminotransferase/ platelet count/ γ-glutamyl transpeptidase/ alphafetoprotein index (APGA)^21^, platelet count/ age/ ALP/ AFP/ AST index (PAPAS)^22^, fibrosis index based on the four factors (King's score)^10^, y-glutamyl transpeptidase/ platelet count ratio index (GPR)^23^, y-glutamyl transpeptidase/ platelet count/ serum albumin index (S-index)^24^, platelet count/ spleen diameter (mm) ratio index (PSR)^25^, monocyte fraction/segmented neutrophil fraction/ platelet count index (P2/MS)^26^ and Platelet count/γ-glutamyl transpeptidase/ age/ cholesterol index (Forns index)^27^ were constructed as described in Table 1.

**Table 1:** Scoring of platelet-based models.

Index	Formulas
Pohl score	$1:\text{AAR}>1\text{and PLT}<\text{150}\times {\text{10}}^{\text{9}}\text{/L or else, the score = 0}$
AARP	$1:\text{AAR}>\text{1 or PLT < 150}\times {\text{10}}^{\text{9}}\text{/L or else, the score}=0$
API	$\begin{array}{c} \text{Age (years):}<\text{30}=\text{0; 30}-\text{39}=\text{1; 40}-\text{49}=\text{2; 50}-\text{59}=\text{3; 60}-\text{69}=\text{4;}\geq \text{70}=\text{5. PLT:}\geq \text{225}=\text{0; 200}-\text{224}\\ =\text{1; 175}-\text{199 = 2; 150}-\text{174}=\text{3; 125}-\text{149}=\text{4;}<\text{125}=\text{5 API is the sum of age and platelet scores and}\\ \text{therefore varied from 0-10} \end{array}$
CDS	$\begin{array}{c} \text{PLT:}>\text{340}=\text{0; 280}-\text{339}=\text{1; 220}-\text{279}=\text{2; 160}-\text{219}=\text{3; 100}-\text{159}=\text{4; 40}-\text{99}=\text{5;}<\text{40}=\text{6 ALT/AST}\\ \text{ratio:}>1.7=\text{0; 1.2}-\text{1.7}=\text{1; 0.6}-\text{1.19}=\text{2;}<\text{0.6}=\text{3 INR:}<\text{1.1}=\text{0; 1.1}-\text{1.4}=\text{1;}>\text{1.4}=\text{2 CDS is the sum}\\ \text{of the above} \end{array}$
APRI	$\text{[AST (/ULN)}\times {\text{100] /PLT (10}}^{\text{9}}\text{/L)}$
FIB-4	$\text{[age (years)}\times \text{AST (U/L)] / [PLT(109/L)}\times {\text{ALT(U/L)}}^{\text{1/2}}]$
FibroQ	10×(Age×AST×PT INR/ALT×PLT)
Lok index	$\text{Log odds}=-5.56-0.0089\times \text{PLT}(10^{9}\text{/L})+1.26\times \text{AAR}+5.27\times \text{INR},\text{lok index}=\exp\;(\log\text{odds})/[1+\exp\;(\log\text{odds})]$
GUCI	$\text{AST}\times \text{INR}\times 100\text{/PLT}(10^{9}\text{/L})$
APGA	$\begin{array}{l} \text{Log}(\text{index})=1.44+0.1490\times \text{log}[\text{GGT}(U/L)]+0.3308\times \log[\text{AST}(U/L)]-0.5846\times \log[\text{PLT}(10^{9}/L)]+0.1148\times \log\\ \;[\text{AFP}(\text{ng}/\text{mL})+1] \end{array}$
PAPAS	$\begin{array}{l} \log(\text{index}+1)=0.025+0.0031[\text{age}(\text{years})]+0.1483\times \log[\text{ALP}(U/L)]+0.004\times \log[\text{AST}(U/L)]+0.0908\times \log\\ \;[\text{AFP}(\text{ng}/\text{mL})+1]-0.028\times \log[\text{PLT}(10^{9}/L)] \end{array}$
King's score	$\text{Age}\times \text{AST}\times {\text{INR/PLT(10}}^{\text{9}}\text{/L)}$
GPR	${\text{GGT/PLT(10}}^{\text{9}}\text{/L)}$
S-index	$\text{1000}\times \text{GGT/[PLT(109/L)}\times {\text{ALB}}^{\text{2}}]$
PSR	${\text{PLT(10}}^{\text{9}}\text{/L)/spleen diameter (mm)}$
P2/MS	${\text{PLT}}^{\text{2}}{\text{(10}}^{\text{9}}\text{/L)/(monocyte fraction}\times \text{segmented neutrophil fraction)}$
Forns index	$\text{7.811}-\text{3.131}\times {\text{ln[PLT(10}}^{\text{9}}\text{/L)] + 0.781}\times \text{ln(GGT) +3.467}\times \text{ln(age)}-0.014\times \text{(cholesterol)}$

AAR, Aspartate aminotransferase/alanine aminotransferase ratio; PLT, Platelet count; AARP, AAR-platelet count score; API, Age/platelet count index; ALT, Alanineaminotransferase; AST, Aspartateaminotransferase; INR, international normalized ratio; CDS, Cirrhosis discriminant score; APRI, Aspartate aminotransferase/ platelet count ratio index; FIB-4, Fibrosis index based on the four factors; PT, Prothrombin time; FibroQ, Fibro-quotient; Lok-index, Fibrosis index based on the three factors; GUCI, Goteborg University Cirrhosis Index; APGA, Aspartateaminotransferase/ platelet count/ y-glutamyl transpeptidase/ alphafetoprotein index; GGT, γ-glutamyl transpeptidase; AFP, Alpha-fetoprotein; ALP, Alkalinephosphatase; PAPAS, Platelet count/ age/ ALP/ AFP/ AST index; King's score, Fibrosis index based on the four factors; GPR, γ-glutamyl transpeptidase/ platelet count ratio index; ALB, serum albumin; S-index, γ-glutamyl transpeptidase/ platelet count/ serum albumin index; PSR, platelet count/ spleen diameter (mm) ratio index; P2/MS, monocyte fraction/ segmented neutrophil fraction/ platelet count index; Forns index, Platelet count/ γ-glutamyl transpeptidase/ age/ cholesterol index.

### Treatment and patient's follow-up

All hypervascular nodules were treated by TACE using oxaliplatin (AiHeng®; Jiangsu HengRui pharmaceutical company limited (co. LTD), Jiangsu, China) or a combination of epirubicin (AiDaSheng®; Hisun Pfizer pharmaceutical co. LTD, ZheJiang, China) or a combination of pirarubicin (THP®; Shenzhen Wanle pharmaceutical co. LTD, Guangdong, China). Patients also performed super-selective catheterization and underwent an injection of Lipiodol into nutrient artery of the tumor, followed by an injection of Collagen Sponge (Trauer®; GuangZhou Trauer Biotechnology Co. LTD, GuangZhou, China). The TACE treatment was repeated every time intrahepatic relapse was found.

Patients were regularly followed at outpatient clinics after discharge, by either CT or MRI, abdominal ultrasound, physical examination, chest radiography, and peripheral blood markers tests (including liver function test and serum AFP). The same evaluations were performed as follow-ups every 3 months for the first year, and every 6 months after the first year. The starting time of follow-up was the date of initial treatment of malignant hepatic tumors. The cut-off time of follow-up was the date of last follow-up (January 2019) or death.

### Statistical analysis

All continuous variables that obey normal distribution were described as the means ± standard deviation and were compared using the independent t-test. Other continuous variables that do not obey normal distribution were presented as the median and range. Univariate analysis of the prognosis and possible clinical factors for categorical variables were tested by Pearson x^2^ test. The overall survival rates were evaluated using the Kaplan-Meier curve, and differences in the survival rates between the groups were statistically compared by the log-rank test. The receiver operating characteristic curve (ROC) was also calculated and the area under the curve (AUC) was generated to discriminate the ability of each scoring systems. Meanwhile, The ROC could obtain the optimal cut-off point (the sum of specificity and sensitivity is the highest cumulative value) of each variable for overall survival. The multivariate analysis was evaluated for the prognostic factors using the Cox proportional hazard model. A bilateral probability (P) value<0.05 was considered statistically significant. All statistical analysis was performed using the IBM SPSS Statistics software package v.23.0 (IBM Corporation, 2015, USA).

## Results

### Patient characteristics

The baseline characteristics of 92 patients are presented in Table 2. The median age of 92 patients was 58 (range 32-82) years. Sixty-seven (72.8%) patients were males and 25 (27.2%) patients were females. One (1.1%) patient was positive for antibodies to hepatitis C virus (anti-HCV), 52 (56.5%) patients were positive for hepatitis B surface antigen. Seventy-five patients (81.5%) had presented normal liver function (Child-Pugh A grade), 49 patients (53.3%) were diagnosed with cirrhosis, and 9 patients (9.8%) presented ascites. All patients were treated by TACE. There was no surgically related mortality. The Pearson x^2^ test indicated that the prognosis of patients with multiple tumor lesions (p=0.032) or presenting vascular cancer embolus (p=0.025) was poor, and there was significant statistical significance.

**Table 2:** Baseline characteristic of included patients.

Variable	Total	Survival status		P value
		Survival	Death	
**Sex**				0.681
man	67	37	30	
women	25	15	10	
**Hepatitis**				0.208
Yes	53	27	26	
No	39	25	14	
**Ascites**				0.442
Yes	9	4	5	
No	83	48	35	
**Cirrhosis**				0.256
Yes	49	25	24	
No	43	27	16	
**Child-Pugh classification**				0.195
A	75	40	35	
B	17	12	5	
**Polycythemia**				0.578
Yes	3	1	2	
No	89	51	38	
**Number of tumors**				
single	53	35	18	0.032
multiple	39	17	22	
**Vascular cancer embolus**				0.025
Yes	16	5	11	
No	76	47	29	
**Age (year)**	57±9	55±9	58±10	0.240
**Diameter of spleen (mm)**	107.25 (77-194.1)	107.25 (83.90-194.1)	106.45 (77-175.3)	0.795
**AFP (ng/ml)**	461.94 (0.83-2000)	238.64 (0.83-2000)	728.20 (1.76-2000)	0.130
**ALB (g/L)**	36.09±5.62	37.60±5.77	35.50±5.52	0.356
**TCH (mmol/L)**	3.62(1.84-8.10)	3.59 (1.94-7.42)	3.89 (1.84-8.10)	0.503
**ALT (U/L)**	45 (7-169)	44.5 (14.4-169)	45.5 (7-120.7)	0.925
**AST (U/L)**	53 (13-438)	51 (13-438)	58 (16-188)	0.262
**ALP (U/L)**	145.50 (63-1854.5)	141.5 (67.1-908)	149.85 (63-1854.5)	0.584
**GGT (U/L)**	153.65 (12-1257)	153.15 (19-1257)	152 (12-622.1)	0.598
**APTT (s)**	33.38±6.45	32.42±6.70	34.53±5.94	0.119
**PT (s)**	12.9 (9.9-17.70)	12.8 (9.9-17.7)	13.45 (11-17.4)	0.464
**INR**	1.08 (0.83-1.50)	1.07 (0.83-1.5)	1.12 (0.92-1.48)	0.419

AFP, alpha fetoprotein; ALB, albumin; TCH, total cholesterol; ALT, alanine transaminase; AST, aspartate amino transferase; ALP, alkaline phosphatase; GGT, gamma-glutamyl transpeptidase; APTT, activated partial thromboplastin time; PT, prothrombin time; INR, international normalized ratio.

The basic characteristics obtained by introducing all parameter values into the 17 platelet-based models were showed in Table 3. The Pearson x^2^ test or Wilcoxon test or independent t-test showed that the Pohl score (p=0.035), CDS (p=0.006), APRI (p=0.032), King's score (p=0.015), Lok index (P=0.015), FIB-4 (p=0.010), FibroQ (p=0.008) and GUCI (P=0.033) were significantly different between survival and death patients.

**Table 3:** Characteristics of platelet count and platelet-based models.

Variable	Total	Survival status		P value
		Survival	Death	
**AARP**				0.447
negative	12	8	4	
positive	80	44	36	
**Pohl score**				**0.035**
negative	55	36	19	
positive	37	16	21	
**PLT** ×**109/L**	136.50(37-447)	149 (38-447)	115.50 (37-322)	0.085
**API**	7(1-10)	6 (2-10)	8 (1-10)	0.074
**CDS**	6(3-10)	6 (3-10)	7 (3-9)	**0.006**
**APRI**	1.16(0.16-16.47)	0.975 (0.22-16.47)	1.596 (0.16-6.6)	**0.032**
**King's score**	27.59(3.68-305.10)	23.27 (4.92-305.10)	34.45 (3.68-225.60)	**0.015**
**Lok index**	0.63(0.09-0.99)	0.56 (0.09-0.97)	0.75 (0.19-0.99)	**0.015**
**P2/MS**	49.88(1.05-521.57)	60.56 (1.05-521.57)	34.14 (1.98-399.04)	0.196
**PAPAS**	3.83±1.26	3.61±1.12	4.10±1.39	0.064
**PSR**	1.29(0.30-4.04)	1.37 (0.30-4.04)	0.99 (0.39-3.40)	0.139
**S-index**	0.84(0.06-10.9)	0.812 (0.10-10.9)	0.892 (0.06-4.54)	0.501
**FIB-4**	3.29(0.87-26.59)	2.867 (0.91-24.50)	4.905 (0.87-26.59)	**0.010**
**FibroQ**	5.66(1.25-45.83)	4.82 1.25-45.83)	7.79 (1.35-45.12)	**0.008**
**GUCI**	48.96(6.23-663.26)	39.21 (8.06-663.26)	62.48 (6.23-300.80)	**0.033**
**GPR**	1.08(0.05-13.66)	1.07 (0.16-13.66)	1.065 (0.05-5.2)	0.671
**APGA**	22.76(4.39-68.33)	22.12 (6.68-65.83)	26.06 (4.39-68.33)	0.097
**Forn index**	10.21±1.91	9.95±1.88	10.53±1.94	0.151

AAR, Aspartate aminotransferase/ alanine aminotransferase ratio; AARP, AAR-platelet count score; P2/MS, monocyte fraction/ segmented neutrophil fraction/ platelet count index; APGA, Aspartateaminotransferase/ platelet count/ γ-glutamyl transpeptidase/ alphafetoprotein index; API, Age/ platelet count index; APR, Aspartate aminotransferase/ platelet count ratio index; PSR, platelet count/ spleen diameter(mm) ratio index; CDS, Cirrhosis discriminant score; S-index, γ-glutamyl transpeptidase/ platelet count/ serum albumin index; Lok-index, Fibrosis index based on the three factors; FIB-4, Fibrosis index based on the four factors; FibroQ, Fibro-quotient; GPR, γ-glutamyl transpeptidase/ platelet count ratio index; GUCI, Goteborg University Cirrhosis Index; PAPAS, Platelet count/ age/ ALP/ AFP/ AST index; King's score, Fibrosis index based on the four factors; Forns index, Platelet count/ γ-glutamyl transpeptidase/ age/ cholesterol index.

### Determining the cut-off value of variables

The ROC curve of PLT showed that 114 × 10^9^/L was an optimum cut-off value, and the sensitivity plus specificity correspondingly was the maximal. Among these models, only CDS (AUC = 0.665, 95/CI: 0.559-0.760), APRI (AUC = 0.632, 95%CI: 0.525-0.730), King's score (AUC = 0.648, 95%CI: 0.542-0.745), Lok index (AUC = 0.647, 95^0^/CI: 0.540-0.744), FIB-4 (AUC = 0.658, 95%CI: 0.552-0.754), FibroQ (AUC = 0.661, 95/CI: 0.555-0.756) and GUCI (AUC = 0.630, 95%CI: 0.523-0.728) were significant indicators for determining prognosis (P < 0.05) (Table 4).

**Table 4:** Comparison of the AUC between PLT-based prognostic scores.

Variable	AUC	95% CI	Cut-off value	P-value
PLT	0.605	0.498-0.705	114×10^9^	0.08
API	0.608	0.500-0.708	4	0.07
CDS	0.665	0.559-0.760	6	0.003
APRI	0.632	0.525-0.730	1.36	0.029
King's score	0.648	0.542-0.745	31.31	0.013
Lok index	0.647	0.540-0.744	0.75	0.013
P2/MS	0.579	0.472-0.681	42.46	0.19
PAPAS	0.607	0.500-0.707	4.36	0.07
PSR	0.590	0.482-0.691	1.04	0.14
S-index	0.541	0.434-0.645	0.64	0.5
FIB-4	0.658	0.552-0.754	5.64	0.008
FibroQ	0.661	0.555-0.756	7.06	0.006
GUCI	0.630	0.523-0.728	60.21	0.03
GPR	0.526	0.419-0.631	0.87	0.7
APGA	0.602	0.494-0.702	32.03	0.09
Forn index	0.602	0.494-0.702	10.57	0.10

AUC, area under the curve; CI, confidence interval; PLT, platelet count; API, Age/ platelet count index; CDS, Cirrhosis discriminant score; APRI, Aspartate aminotransferase/ platelet count ratio index; King's score, Fibrosis index based on the four factors; Lok-index, Fibrosis index based on the three factors; P2/MS, monocyte fraction/ segmented neutrophil fraction/platelet count index; PAPAS, Platelet count/ age/ ALP/ AFP/ AST index; PSR, platelet count/ spleen diameter(mm) ratio index; S-index, γ-glutamyl transpeptidase/ platelet count/ serum albumin index; FIB-4, Fibrosis index based on the four factors; FibroQ, Fibro-quotient; GUCI, Goteburg University Cirrhosis Index; GPR, γ-glutamyl transpeptidase/ platelet count ratio index; APGA, Aspartateaminotransferase/ platelet count/ γ-glutamyl transpeptidase/ alphafetoprotein index; Forns index, Platelet count/ γ-glutamyl transpeptidase/ age/ cholesterol index.

### Survival and prognostic factors

The median time of follow-up was 32.5 (range 1-73) months. Fifty-two (56.5%) patients were alive at the end of the follow-up period, and 40 (43.5%) patients had died. The 1-year, 3-year and 5-year overall survival rates were 83.5%, 64.5%, and 40.2%, respectively.

**Figure 1:**
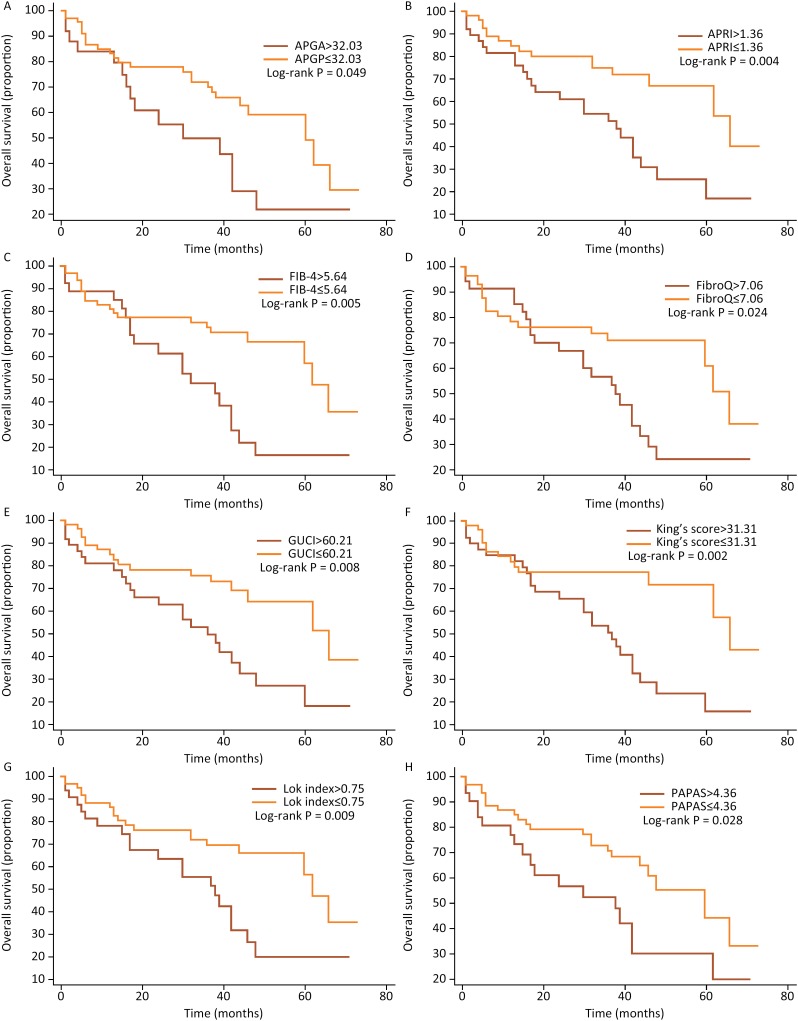
Overall survival. Kaplan-Meier curves of patients stratified according to **A**) APGA, **B**) APRI, **C**) FIB-4, **D**) FibroQ, **E**) GUCI, **F**) King's score, **G**) Lok index and **H**) PAPAS.

According to the optimum cut-off value determined by The ROC curve, the continuous variables of platelet-based scores were divided into two categories. The relationship between the platelet-based prognostic scores and overall survival is shown in Figure 1. The APGA>32.03, APRI>1.36, FIB-4>5.64, FibroQ>7.06, GUCI>60.21, King's score>31.31, Lok index>0.75 and PAPAS>4.36 were associated with a reduced overall survival (all P<0.05). Meanwhile, a log-rank analysis demonstrated that patients with cirrhosis, multiple tumor, vascular cancer embolus, AFP> 8.54 ng/mL, ALP>146U/L and APTT>28.1s had a reduced probability of postoperative survival (Table 5).

**Table 5:** Log-rank test of possible clinical factors associating with survival status.

Variable	P value
Sex (male/female)	0.396
Age (≤52Y/>52Y)	0.112
Cirrhosis	0.049
HBV (Yes/No)	0.171
Ascites (Yes/No)	0.337
Polycythemia (Yes/No)	0.573
Number of tumors (single/multiple)	0.040
Vascular cancer embolus (Yes/No)	<0.001
AFP(≤8.54ng/ml/>8.54ng/ml)	0.038
ALT(≤98U/L/>98U/L)	0.316
AST(≤38U/L/>38U/L)	0.058
ALP(≤146U/L/>146U/L)	0.016
GGT(≤177U/L/>177U/L)	0.415
ALB(≤40g/L/>40g/L)	0.105
PT(≤13.3s/>13.3s)	0.107
APTT(≤28.1s/>28.1s)	0.001
INR(≤1.11/>1.11)	0.131
Diameter of spleen(≤ 95.3mm/>95.3mm)	0.082
PLT×10^9^(≤114/>114)	0.132
APGA(≤32.03/>32.03)	0.049
API(≤4/>4)	0.148
APRI(≤1.36/>1.36)	0.004
CDS(≤6/>6)	0.088
FIB-4(≤5.64/>5.64)	0.005
FibroQ (≤7.06/>7.06)	0.024
Forn index(≤10.57/>10.57)	0.162
GPR(≤0.87/>0.87)	0.056
GUCI(≤60.21/>60.21)	0.008
King's score(≤31.31/>31.31)	0.002
Lok index(≤0.75/>0.75)	0.009
P2/MS(≤42.46/>42.46)	0.126
PAPAS(≤4.36/>4.36)	0.028
PSR(≤1.04/>1.04)	0.193
S-index(≤0.64/>0.64)	0.200
Pohl score(negative/positive)	0.166
AARP(negative/positive)	0.914

AFP, alpha fetoprotein; ALT, alanine transaminase; AST, aspartate amino transferase; ALP, alkaline phosphatase; GGT, gamma-glutamyl transpeptidase; ALB, albumin; PT, prothrombin time; APTT, activated partial thromboplastin time; INR, international normalized ratio;. PLT, platelet count; APGA, Aspartateaminotransferase/ platelet count/ γ-glutamyl transpeptidase/ alphafetoprotein index; API, Age/ platelet count index; APRI, Aspartate aminotransferase/ platelet count ratio index; CDS, Cirrhosis discriminant score; FIB-4, Fibrosis index based on the four factors; FibroQ, Fibro-quotient; Forns index, Platelet count/ γ-glutamyl transpeptidase/ age/ cholesterol index; GPR, γ-glutamyl transpeptidase/ platelet count ratio index; GUCI, Goteborg University Cirrhosis Index; King's score, Fibrosis index based on the four factors; Lok-index, Fibrosis index based on the three factors; P2/MS, monocyte fraction/ segmented neutrophil fraction/ platelet count index; PAPAS, Platelet count/ age/ ALP/ AFP/ AST index; PSR, platelet count/ spleen diameter(mm) ratio index; S-index, γ-glutamyl transpeptidase/ platelet count/ serum albumin index;. AAR, Aspartate aminotransferase/alanine aminotransferase ratio; PLT, Platelet count; AARP, AAR-platelet count score.

Multivariate analysis of clinical characteristic variables expressed as binary variables showed that the number of tumors, ALP and APTT were independent risk factors for overall survival. Among the noninvasive platelet-based scores, multivariate analysis for the 17 predictive factors and platelet count revealed that APGA (HR 3.213, 95% CI 1.089-9.477, P = 0.034) was independently associated with overall survival. The other 16 platelet-unrelated scores and platelet count, API, APRI, CDS, FIB-4, FibroQ, Forn index, GPR, GUCI, King's score, Lok index, P2/MS, PAPAS, PSR, S-index, Pohl score and AARP, were not independent risk factors for overall survival. (Table 6).

**Table 6:** Results of multivariate analyses for survival status.

Variable	OR	95%CI	P
Cirrhosis	1.272	0.558-2.901	0.568
Number of tumors	0.52	0.258-1.048	0.067
Vascular cancer embolus	0.219	0.084-0.570	0.002
AFP	0.513	0.176-1.496	0.221
ALP	0.406	0.179-0.922	0.031
APTT	0.088	0.021-0.371	0.001
APGA	3.213	1.089-9.477	0.034
APRI	0.558	0.094-3.297	0.52
FIB-4	0.500	0.104-2.414	0.388
FibroQ	2.037	0.440-9.431	0.363
GUCI	1.472	0.245-8.833	0.672
King's score	0.460	0.116-1.824	0.269
Lok index	0.666	0.278-1.591	0.360
PAPAS	0.923	0.344-2.481	0.874

OR, odds ratio; CI, confidence interval; AFP, alpha fetoprotein; ALP, alkaline phosphatase; APTT, activated partial thromboplastin time; APGA, Aspartateaminotransferase/ platelet count/ γ-glutamyl transpeptidase/ alphafetoprotein index; APRI, Aspartate aminotransferase/ platelet count ratio index; FIB-4, Fibrosis index based on the four factors; FibroQ, Fibro-quotient; GUCI, Goteborg University Cirrhosis Index; King's score, Fibrosis index based on the four factors; Lok-index, Fibrosis index based on the three factors; PAPAS, Platelet count/age/ ALP/ AFP/ AST index.

## Discussion

The ultimate goal of a treatment for malignant hepatic tumors was to prolong survival time by curative therapy. TACE and resection were the most frequent preferred treatments for advanced hepatocellular carcinoma in clinical practice^28^. When patients couldn't perform radical resection, TACE treatment might be a good choice for these patients. For example, a single central lesion that located between the portal vein and the inferior vena cava might be an indication of TACE treatment, the reason was the difficulty of radical resection or the insufficient volume of residual liver. However, indications for TACE were not applicable to all candidates such as Child-Pugh C grade. For Child-Pugh C grade, it could be restored to Child-Pugh A or B grade after adjustment, and TACE treatment could still be performed. This didn't mean that all patients with advanced HCC could benefit from TACE treatment. The variation in prognosis was significant according to the number of prognostic risk factors, with median survival time from 5.5 months to 21.4 months^29^. Thus, it was crucial to determine the predisposing factors for prognosis and improve them before treatment. The roles of platelet count and platelet-based score models for assessing such risk factors were emphasized in this retrospective analysis of 92 patients. The results confirmed that the number of tumors, ALP and APTT were independent prognostic factors of overall survival. We also determined APGA as independent predictors of overall survival after TACE therapy.

Platelet usually released all kinds of cytokines to participate in the inflammatory response, such as platelet-derived growth factors and transforming growth factor p. Platelets also transport these substances to specific positions, platelet played an important role in angiogenesis, wound healing, liver regeneration and so on^7^. Most liver cancers were accompanied with cirrhosis, which was mainly caused by chronic hepatitis. When liver cancer forms, cancer cells could disorder the balance of the blood coagulation system by producing high levels of blood coagulation factors. Besides, the disorders of the coagulation system promoted excessive platelet activation. The activated platelets could provide the procoagulant surface to induce cancer-related gather. Meanwhile, the platelets were recruited to surround tumor cells, which protected them from the body's immune detection and promoted the growth and metastasis of the cancer^30^ ^,^ ^31^. It could also promote the release of active medium of platelets and increase the permeability of blood vessels to achieve the goal of cancer proliferation^32^. Therefore, these theoretical backgrounds had led to the proposal of several platelet-based prognostic scores in patients with cancer over the last 10 years.

Several studies had demonstrated that PLT was associated with hepatocellular carcinoma, and several platelet-based models had been determined as predictors of cancer formation^33^. Tamaki et al reported that the FIB-4>3.25 independently increased the risk of developing hepatocellular carcinoma by a factor of 1.7^34^. Amano *et al.* ^35^ demonstrated that low-level PLT was associated with a higher risk of recurrence in hepatocellular carcinoma. Many prognostic models of malignant hepatic tumors had been reported, but which model was more suitable for predicting the prognosis of malignant hepatic tumors was still inconclusive. Moreover, reliable indices to predict long-term prognosis after TACE were still scarce. To improve the outcomes of advanced malignant hepatic tumor patients after TACE, it was necessary to illustrate the mechanism of oncogenesis and explore the clinically crucial risk factors associated with long-term prognosis, which might be the potential therapy targeting except for radical surgery. In this study, we believed that platelet count and platelet-based scores might be potential therapeutic targets and the score of APGA>32.03 was an independent risk factor associated with poor overall survival for patients with malignant hepatic tumor undergoing TACE. Therefore, the APGA index would be best applicable in predicting prognosis, and overall survival also could be predicted according to APGA score and corresponding preoperative adjustment could be made.

The result was consistent with that of Fung et al, the APGA score could predict hepatitis, cirrhosis, even liver cancer^21^. As can be seen from Figure 1A, the higher the score, the lower the survival rate after TACE. According to the calculation formula of APGA from Table 1, the higher the platelet count, the higher the score. The platelet count was not an independent risk factor for prognosis but the APGA score was; the reason for this result was the combined effect of PLT, AST, GGT and AFP. Although the AUC value of APGA score (AUC=0.602) was not the largest in Table 4 and the P value is greater than 0.05, it did not mean that the APGA score was worthless. The main purpose of table 4 was to obtain the optimal cutoff value by ROC curve, rather than to compare various platelet models to diagnose patient outcome events. As long as the AUC value of APGA was more than 0.5, we could still perform further analysis and research on the APGA model.

This study was an exploratory analysis of clinical data including patients who underwent TACE treatment and obtained different prognosis backgrounds. In this study, in addition to the conclusion that APGA was an independent risk factor affecting the prognosis after TACE, the number of tumors, ALP and APTT were also independent risk factors. Chan et al pointed out that the number of tumors was a key parameter for early recurrence of HCC^36^. Our results in line with Chan, multi-nodulose tumor was associated with poor prognosis after TACE. We believed that multi-nodulose tumor might have undetected micro lesions, which were easy to recurrence after surgery. Coagulation disorders and abnormal liver metabolism were usual complication in liver diseases, especially advanced tumors. Our results also suggested that ALP and APTT were independent risk factors for prognosis after TACE. Therefore, we can also predict the prognosis of TACE by ALP and APTT preoperatively.

A potential limitation of this study was that was a retrospective, single-center study. Therefore, a large- scale prospective study was needed to validate the result. These scores were not compared with platelet- unrelated indices and stratification analysis of patients was not used to determine whether results of platelet- based models were affected.

This study was the first to explore the performances of 17 platelet-based scores for preoperative detecting overall survival after TACE in malignant hepatic tumors. Furthermore, we could make appropriate preoperative adjustment according to the score of the optimum platelet-based model, so as to improve the survival rate and prolong survival time after TACE. Additionally, all selected platelet-based models had been reported and accepted that have been repeatedly validated in corresponding liver diseases. Taken together, the study showed that APGA might be an effective tool to assess postoperative prognosis in patients with malignant hepatic tumors, especially overall survival. As the prognostic index was noninvasive, inexpensive, and easy to count, these findings would be meaningful for surgeons or interventional radiologists assessing postoperative overall survival in malignant hepatic tumors.

## Conclusion

The APGA, a platelet-based prognostic score, was an independent marker of prognosis in patients with malignant hepatic tumors after TACE and was superior to the other platelet-based prognostic scores in terms of prognostic ability.
